# Self-care practice and associated factors among epileptic patients: a cross-sectional study, Ethiopia

**DOI:** 10.11604/pamj.2023.44.36.31554

**Published:** 2023-01-18

**Authors:** Ismael Ahmed, Abebe Abera, Tigist Demeke, Gemechu Terefe, Sheka Shemsi, Abduwalhid Awol

**Affiliations:** 1 Jimma University, Institute of Health, Faculty of Health Science, School of Nursing and Midwifery, Addis Ababa, Ethiopia

**Keywords:** Epilepsy, self-care practice, epileptic patients

## Abstract

**Introduction:**

epilepsy results in multidimensional and long term effect on the patients and society. Self-care practice is critical for epileptic patient. So far, the issue of self-care practice still considered as the most important cause of poorly controlled epilepsy. Yet comprehensive epilepsy self-care practice is not recognized, which is not addressed with medical treatment alone has not been studied particular in Ethiopia. The objective was to assess self-care practice and associated factors among epileptic patients on follow up at Jimma Town public hospitals, 2020.

**Methods:**

institution based cross-sectional study was conducted from April 08 - May 20/2020. Data was collected using structured interviewer administered questionnaire and data extraction checklist. Simple random sampling technique was used to select a total of 297 study participants. Data was entered to EPI data version 3.5.3 and exported to SPSS version 23.0 for analysis. Variables with p-value < 0.25 on bivariate analysis were candidated for multivariate analyses. Factors with p value < 0.05 on multivariate analyses were considered as statistically significant.

**Results:**

a total of 297 study participants were included in the study giving a response rate of 99.0%. Of study participants 146(49.2%) of them were had good self-care practice. Residence (AOR= 1.712, 95%CI: 1.034-2.836, P- 0.037), Seizure frequency (AOR = 0.288, 95% CI: 0.091-0.907, P-0.034), felt stigma (AOR=0.565, 95%CI: 0.342-0.935, P- 0.026) and medication adherence (AOR=0.391, 95%CI: 0.240-0.638, P-0.000) were significantly associated with self-management practice.

**Conclusion:**

this study found that half of the study participants were had poor self-care practice. Residence, felt stigma, increased seizure frequency and not adherence to medication were factors contributed for poor self-care practice. Therefore, intervention strategies focused on contributing factor for poor self-care practice should be considered.

## Introduction

Epilepsy is the most common chronic neurological disorder characterized by recurrent and unprovoked seizure, affecting 50 million worldwide [1]. Epilepsy results in variety of medical, social, psychological and economic impact particularly in developing countries in which its incidence and prevalence is highly increasing [2]. In Africa around 10 million people are directly affected by epilepsy and in addition to its high prevalence there is a wide treatment gap in Africa. Epilepsy is the second most burdensome neurologic disorder worldwide in terms of disability-adjusted life years and with lifetime prevalence of 7.6 per 1,000 persons. Worldwide, mortality of epilepsy is two to three times higher than in the general population and it is thought to be higher in developing countries particularly in sub-Saharan Africa [1-3]. In Ethiopia prevalence of epilepsy was 5.2/1000 populations with annual incidence of 64 per 100,000 populations [3]. Self-care practice is critical part of patient and family centered care. Components of epilepsy self-management include medication management, information management, safety management, seizure management and lifestyle management [4-6]. Good epilepsy self-care encompasses a set of skills including good medication adherence, being able to accurately describe and document one's seizures, practicing safety precautions, having adequate rest, and managing one's stress levels [5,6].

Patient´s self-care practice has invaluable role in controlling the illness, to minimize its impact on health and quality of life, and to cope with the disease. In addition, self-care enables patient to develop appropriate skills and build confidence in implementing health behavior to better management of a condition and increase their self-efficacy in their day-to-day activities and lead better quality lives [4,7,8]. Furthermore, encouraging patient on self-care practice can reduce health care expenditure and lower utilization of health care services [6]. Though self-care practice plays an important role in comprehensive epilepsy care, and improving clinical outcomes, it has been reported that epileptic patients are less committed to practice in improving the management of their disease [9,10]. Lack of practicing of health-promoting self-management behaviors can potentially decrease patient´s self-confidence and ability to manage disease [10]. Studies reported social stigma, insufficiency of social support or experience social isolation and poorly controlled seizures are contributed for patients´ less commitment to self-management practice [2,10,11]. Most of previous studies done in Ethiopia mainly focused on medication related management of epilepsy [3,12] and, there is no data regarding epileptic patient´s self-care practice so far. Therefore, present study aimed to assess self-care practices and associated factors among epileptic patients having long term follow up at public hospitals in Jimma town, Southwest Ethiopia.

## Methods

**Study settings, study design, study period and study participant:** the study was conducted at two public hospitals of Jimma town (Jimma University Medical Center (JUMC) and Shenan Gibe Hospital (SGH)). JUMC is the oldest public hospital in the country and it is the only referral hospital in the south western part of the country providing service for more than 18 million people. More than 12,384 and 5832 patients had follow up at chronic clinic of JUMC and SGH respectively. Of them around a total of 750 and 370 were epileptic patients having follow up at JUMC and SGH respectively. Institution based cross-sectional study was conducted from April 08- May 20/2020. Epileptic patients aged 18 years and above on follow up at chronic clinic of JUMC and SGH were included in the study and patient with incomplete medical record and inability to communicate were excluded.

**Sample size determination and sampling technique:** the sample size was calculated using single population proportion formula as follows:


$$n=\frac{Z\left(\frac{\alpha }{2}\right)2\text{P}\left(I-P\right)}{d^{2}}$$

Where n is minimum sample size. P is prevalence of self-care practice. d2 is margin of error (5%) and Z a/2 is the 95 % confidence interval. By taking prevalence of self-care practice 50% since no previous study on this particular topic and margin of error (5%) and substituting the values for each variables in single proportion formula n = (1.96)^2^ 0.5 (0.5-1)/ (0.05)^2^ = 384. Since source population for this study is less than 10,000 the correction formula was used:


$$\text{nf}=\frac{\text{nc}}{1+\text{nc/N}}$$

Where: nf is final sample size. nc is calculated sample size. N is source population (all adult epileptic patients on follow up at both hospitals). Substituting the values for each variables nf = 384/ 1+384 / 1120 = 286. By considering 5% non-response rate, total sample size was 286+14= 300. Sample size was proportionally allocated for both hospitals (JUMC, N=750, n = 201 and SGH, N=370, n=99). Simple random sampling technique was used to select the study subjects by taking patients medical records as randomization unit.

**Data collection procedures and instrument:** structured interviewer administered questionnaire and data abstraction checklist was used to collect data. The questionnaire contains five parts: socio-demographic variables, clinical and treatment related factors, psychosocial variables, Morisky 8-item medication adherence scale and epilepsy self-management scale.

**Adherence to medication** was measured by an eight-item Morisky Medication Adherence Scale (MMAS) [12]. Items 1-7 are yes/no questions, in which a “no” answer receives a score of 1 and a “yes” answer receives a score of 0, except for item 5, which is reverse-scored. Item 8 is measured on a five-point scale. The responses “never”, “once in a while”, “sometimes”, “usually”, and “all the time” are scored, 1, 0.75, 0.50, 0.25, and 0, respectively. Adherence to medication was considered as low, medium, and high adherence if the total score is < 6, 6 to < 8, and 8 points, respectively. In this particular study individual in category of medium adherence and high adherence are taken as adherent and low adherence as non-adherent. Felt stigma was assessed with the Jacoby scale (JS) composed of three questions [13]. Each item was a Boolean question (no = 0, yes = 1). The score of the JS was established from the sum of each item. The sum score >0 indicated presence of felt stigma and sum score = 0 indicates not felt stigma. Social support was assessed by using the Oslo 3-items social support scale in which the first item had 4- point Likert scale and the other two items had 5-point Likert scale [12]. The sum score scale range from 3-8, 9-11, and 12-14 indicates poor, moderate and strong support respectively.

**Patient´s beliefs about medication** were assessed using the belief about medicines questionnaire (BMQ) [3]. It contains 18 questions in two parts (general beliefs and specific beliefs about drug). In this study specific belief about the drug which contains two parts, specific- necessity which assess patients´ belief about the necessity of the prescribed medications for controlling their illness and **specific- concerns** about the potential adverse consequences of taking medication was used. Each question was scored based on 5 point Likert scale (1-Strongly disagree, 2-Disagree, 3- No comments, 4-Agree, 5- Strongly agree). Accordingly, participants were considered to have strong medication necessity belief if the average sum of the five-item medication necessity scale score (ranging from 5-25) is mean and above. Conversely, if the score is below mean they were considered to have low medication necessity belief. Similarly, participants were considered to have strong concern belief about their medication adverse effect if the average sum of the five-item medication concern scale score (ranges from 5-25) is mean and above, otherwise, they were considered to have low medication concern belief.

**Self-care practice** was measured by 38 item epilepsy self-management scale contains five major domains (MM, IM, SM, SEM and LM) [14]. Each question was scored based on 5 point Likert scale (1- Never, 2- Rarely, 3- Sometimes, 4- Most of the Time, 5- Always). Total scores found by reverse coding the 12 negatively worded items and summing responses to all 38 individual items. Total possible scores range from 38-190.

**Operational definition:** participants who scored mean and above of Medication, Information, Safety, Seizure and Lifestyle management questioner´s domain of ESMS were considered as had good Medication, Information, Safety, Seizure and Lifestyle management practice while those score less than mean were considered as had poor Medication, Information, Safety, Seizure and Lifestyle management practice respectively. The overall self-care practice of participants was considered as Good self-care practice when their overall ESMS score is mean and above and Poor self-care practice when their overall ESMS score is below mean.

**Data collection technique and data quality control:** data was collected through face to face interview and reviewing of patient´s medical records. Data was collected by trained four B.Sc. nurse and two MSc nurses were assigned as supervisors. Pre-test was done on 5% of the sample size, at Agaro hospital two weeks before actual data collection and based on the finding of the pretest the questioner was revised.

**Data analysis:** the collected data was checked for completeness, cleaned and entered in to Epi data version 3.5.3 software and exported to SPSS version 23.0 software for analysis. Descriptive statistics were used to describe the data. Variables with p-value < 0.25 on bivariate analysis were candidated for multivariate analyses. Factors with p value < 0.05 on multivariate analyses were considered as statistically significant.

**Data availability:** the dataset of this article is accessible on reasonable request from the corresponding author.

**Source of funding:** this study was funded by Jimma University, Ethiopia and the funder has no interference with the conduction, analysis and publication process.

**Ethical clearance and consent to participate:** ethical clearance was obtained from the Institutional Review Board (IRB) of Jimma University, Institute of Health, and Faculty of Health Science. Verbal consent was obtained from the respondents (because of the title of the study is not expose the participants for risk and the study have no experiment) and was approved by the Institutional Review Board (IRB) of Jimma University. All information obtained from the patients and records were kept confidential.

## Results

**Demographic characteristics:** of calculated 300 sample a total of 297 study participants were included in the study. From a total of the study participants, 169 (56.9%) of them were male. Almost half 145(48.8%) of the study participants were in the age category of 18-25 years (Table 1).

**Table 1:** socio-demographic characteristics of study participants on follow up at Jimma Town public hospitals, Ethiopia, 2020 (n=297)

Variables	Categories	Frequency (N)	Percent (%)
Age in years	18-25	145	48.8
	26-33	70	23.6
	34-41	35	11.8
	42-49	17	5.7
	>=50	30	10.1
Religion	Muslim	191	64.3
	Orthodox	66	22.2
	Protestant	31	10.4
	Others	9	3.0
Occupational Status	Government employee	33	11.1
	Farmer	111	37.4
	Merchant	34	11.4
	Daily labor	21	7.1
	Student	73	24.6
	House wife	25	8.4
Marital status	Single	142	47.8
	Married	133	44.8
	Divorced	12	4.0
	Widowed	10	3.4
Educational status	Illiterate	95	32.0
	Grade 1-8	119	40.1
	Grade 9-12	53	17.8
	College and above	30	10.1
Residence	Rural	106	64.3
	Urban	191	35.7
Monthly income In Ethiopian birr	<500	175	58.9
	500-1000	31	10.4
	>1000	91	30.6

* Religion, Others: - waqefata, Adventist and Catholic

**Clinical and treatment characteristics of study participants:** majority, 222 (74.7 %) of the study participants had generalized tonic clonic seizures. More than one third of the respondents 110 (37.0%) had treatment for 6-10 years. About 73.1% of the respondents were experiencing less than two episodes of seizure since their last visit (Table 2).

**Table 2:** frequency distribution of clinical and treatment characteristics of study participants on follow up at Jimma Town public hospitals, Ethiopia, 2020 (n=297)

Variables	Categories	Frequency (N)	Percent (%)
Duration of the disease in year	<1	17	5.7
	1-5	78	26.3
	6-10	74	24.9
	>10	128	43.1
Duration on AED treatment in year	<1	49	16.5
	1-5	110	37.0
	6-10	83	27.9
	>10	55	18.5
Treatment regimen	Mono therapy	172	57.9
	Poly therapy	125	42.1
Current AEDs	Phenobarbital	185	62.3
	Phenytoin	87	29.3
	Sodium-valproate	13	4.4
	Carbamazepine	8	-
	Valporpic acid	4	-
History of seizure since last visit	Yes	80	26.9
	No	217	73.1
Seizure frequency since last visit	<=2 episode	277	93.3
	>=3 episodes	20	6.7
Frequency of doses per day	Once daily	129	43.4
	Twice daily	168	56.6
Way of Getting medication	Freely	184	62.0
	Payment	113	38.0
Presence of Comorbid illness	Yes	80	26.9
	No	217	73.1

**Psychosocial factors of study participants:** from total of study participants more than one third 109(36.7%) of them were felt stigmatized. Almost half 143 (48.1%) of the respondents had strong medication concern belief (Table 3).

**Table 3:** frequency distribution of psychosocial factors among study participants on follow up at Jimma Town public Hospitals, Ethiopia, 2020 (n=297)

Variables	Categories	Frequency (N)		Percent (%)
Felt stigma	Not felt stigmatized	188		63.3
	Felt stigmatized	109		36.7
	Total	297		100.0
Social support	Poor social support	119		40.1
	Moderate social support	133		44.8
	Strong social support	45		15.2
	Total	297		100.0
Belief about medication	Specific concern	Strong medication concern belief	143	48.1
		Low medication concern belief	154	51.9
		Total	297	100.0
	Specific necessity	Strong medication necessity belief	220	74.1
		Low medication necessity belief	77	25.9
		Total	297	100.0
Medication adherence	Adhere	50.8		151
	Not adhere	49.2		146
	Total	100		297

**Components and overall epilepsy self-care practice:** the mean of life style, seizure, self-management, medication and information management practice were 17.6, 19.7, 29.5, 40.4 and 14.8 respectively. Of study participants 146 (49.2%) of them were had good overall epilepsy self-care practice (Table 4).

**Table 4:** frequency distribution of components of epilepsy self-care among study participants on follow up at Jimma Town public hospitals, Ethiopia, 2020 (n=297)

Component of self-management practice	Categories	Frequency (N)	Percent (%)
Life>	Good life>	156	52.5
	Poor life>	141	47.5
Seizure management	Good seizure management practice.	174	58.6
	Poor seizure management practice.	123	41.4
Self-management	Good self-management practice.	151	50.8
	Poor self-management practice.	146	49.2
Medication management	Good medication management practice.	157	52.9
	Poor medication management practice.	140	47.1
Information management	Good information management practice.	135	45.5
	Poor information management practice.	162	54.5
**Overall self-care practice**.	Good self-care practice,	146	49.2
	Poor self-care practice.	143	50.8

**Multivariate analysis of factors associated with self-care practice:** during multivariate analysis of self-care practice in relation to the candidate variables for multivariate analysis, residence, seizure frequency, felt stigma and medication adherence were significantly associated with self-care practice (Table 5).

**Table 5:** factors associated with self-care practice among study participants on follow up at Jimma Town public hospitals, 2020 (n=297)

Variable	Category		Overall self-care practice		COR	AOR	95%CI		p-value
			Good self-care practice	Poor self-care practice			lower	higher	
Sex	Male		76(45.0)	93(55.0)	1	1			0.703
	Female		70(54.7)	58(45.3)	0.098	.903	.535	1.524	
Educational status	Illiterate		44(46.3)	51(53.7)	1	1			0.271
	Primary (1-8)		54(45.4)	65(54.6)	1.014	1.333	.740	2.400	0.338
	Secondary (9-12)		34(64.2)	19(35.8)	1.053	.667	.305	1.459	0.311
	College and above		14(46.7)	16(53.3)	.489	1.324	.499	3.514	0.573
Residence	Rural		60(56.6)	46(43.4)	1	1			0.037*
	Urban		86(45)	105(55)	0.057	1.712	1.034	2.836	
Seizure frequency	<=2 episode		103(46.9)	107	1	1			**0.034**
	3 and above episode		16(80)	4(20)	0.007	.288	.091	.907	
Disease duration	< 1 year		4(25)	12(75)	1	1			0.342
	1-5 year		41(51.9)	38(48.1)	2.565	.506	.141	1.822	0.298
	6-10 year		42(56.8)	32(43.2)	.793	.406	.112	1.472	0.170
	>10 year		59(46.1)	69(53.9)	.651	.663	.191	2.296	0.516
Treatment duration	< 1 year		22(44.9)	27(55.1)	1	1			0.317
	1-5 year		63(57.3)	47(42.7)	.882	1.016	.413	2.500	0.973
	6-10 year		38(45.8)	45(54.2)	.536	2.000	.669	5.976	0.215
	>10 year		23(41.8)	32(58.2)	.851	1.602	.491	5.226	0.434
Stigma	Not felt stigmatized		81(43.1)	107(56.9)	1	1			0.026*
	Felt stigmatized		65(59.6)	44(41.4)	0.006	.565	.342	.935	
Social support	Poor		66(55.5)	53(44.5)	1	1			0.118
	Moderate		56(42.1)	77(57.9)	.918	1.537	.891	2.651	0.122
	Strong		24(53.3)	21(46.7)	1.571	.784	.364	1.688	0.534
Medication Believes	Concern	Strong	76(53.1)	67(46.9)	1	1			0.743
		Low	70(45.5)	84(54.5)	0.186	1.095	.637	1.881	
Medication adherence	Adhere		55(36.4)	96(63.6)	1	1			0.000*
	Not adhere		91(62.3)	55(37.7)	0.000	.391	.240	.638	

## Discussion

Self-care practice is vital in preventing or minimizing the impact of disease on everyday life. This cross-sectional study assessed the status self-care practice among epileptic patients. The finding of this study shows 49.2% of study participants had good self-care practice, which is lower than a cross sectional study conducted at tertiary care hospitals of Mangalore south India which reports that 71.4% of study participant had good self-care practices [9]. The discrepancy in the finding may be due to difference in the study period, selection of participants and sample size in which cited study conducted eight years back to this particular study and includes participants above 7 years and a total of 56 participants. It also might be due to the difference in operationalizing of status of self-care practice in which cited study focus only on same specific components ESMS to classify self-care practice status of participants.

This study shows seizure management practice was significantly higher than information management practice while, life style, self-management and medication management practice were almost in similarly status which is supported by study conducted in USE in which mean score of seizure management practice was significantly higher than information management [6]. The consistency in the finding might be due to, patient may fear events of episodes of seizures and they try to avoid the situation that provokes seizure. The finding of this particular study shows that residence of participants was significantly associated with self-care practice. This might be due to, study participants from urban may have more accesses to health care professional advice, information about disease and precautions to control disease. In this study, felt stigmatized was significantly associated with self-care practice. It is supported by study conducted in USE, revealed felt stigma were significantly associated with self-care practice [15]. This might be due to, the study participants fear to practice self-care regularly in the presence of others due to fear of judged or ignored by others. In this study medication adherence was significantly associated with self-care practices which in line with study conducted at north England, indicate there were significant differences between those adherents to drug and non-adherent on self-care practice [16]. It might be due to the medication management component of ESMS related to adherence to prescribed medication contributed to the overall epilepsy self-care practice.

This particular study shows that frequency of seizure was significantly associated with self-care practice which is supported by the study conducted at Ohio State University Medical Center´s that revealed increased frequency of seizure significantly affects patients´ self-care practice [17]. Participants experiencing more episodes of seizure may hesitate the effectiveness prescribed medications and other precautions on the control of seizure and may loss hope on the progress of disease. The findings of this project provide policymakers and healthcare planners in Ethiopia with evidence of research to design the strategies to improve self-care practice of epileptic patients. The finding of this study would give insight and enable healthcare administrators to promote and strengthen self-care practice of epileptic patients in patient education plan. Furthermore, this study serves as a benchmark and provides baseline primary data for further research on self-care practice in epileptic patients in Ethiopia and other countries in the future. This particular study was limited to hospitals founds in Jimma town, which might not be generalized to total population across the countries. Using face-to-face interview may lead to social desirability bias by over-estimating or under-estimating the result. This study was conducted using a cross-sectional study design in which the link between the outcome and the exposure cannot be determined because of the exposure and outcome was simultaneously assessed.

## Conclusion

Even though self-care practice is an important component of epilepsy treatment, this study found that self-care practice is poor, which highlights the prevalent problematic degree of self-care practice. So that encouraging the patients on the importance of self-care on control of disease should be considered as routine part of epilepsy management. Information dissemination to the people with epilepsy and to the public at large is important to improve self-care practice and to promote healthy life for those individuals.

### What is known about this topic

- *Epilepsy results in multidimensional and long-term effect on the patients and society;*
- *Self-care practice is critical for epileptic patient so far, the issue self-care practice still considered as the most important cause of poorly controlled epilepsy;*
- *Epilepsy medical treatment alone has been studied*.

### What this study adds

- *Comprehensive epilepsy management including epilepsy selfcare practice is studied;*
- *Almost half of the study participants had good self-care practice;*
- *Residence, seizure frequency, felt stigma and medication adherence were significantly associated with self-management practice*.
